# The prospective Austrian hypertrophic cardiomyopathy registry – design, methods and results of the run-in period

**DOI:** 10.1007/s10741-026-10630-6

**Published:** 2026-04-15

**Authors:** Viktoria Santner, Christina Granitz, Martin R. Grübler, Daniel Dalos, Christian Reiter, Marc-Michael Zaruba, Johann Auer, Deddo Moertl, Anna Rab, Peter P. Rainer, Gert Waltl, Christian Ebner, Thomas Weber, Diana Bonderman, Stephan Dobner, Hannah Tuppinger, Viktoria Höller, Nora Schwegel, Klemens Ablasser, Andreas Zirlik, Amelie Graf, Sarah Gharibeh, Johannes Lanzerstorfer, Katharina Wörgötter, Christopher Mann, Shehroz Masood, Christy Meledeth, Clemens Steinwender, Andre Logtenberg, Moritz Messner, Carina Primus, Pia Auersperg, Lydia Mackova, Christof Baurecht, Susanne Winkler, Stephanie Schneiderbauer-Porod, Kathrin Danninger, Silvia Charwat-Resl, Paul Harbich, Rene Krenn, Nicolas Verheyen

**Affiliations:** 1 Department of Cardiology, University Heart Center Graz, Medical University of Graz, Auenbruggerplatz 15, 8036 Graz, Austria; 2 Clinic of Internal Medicine II, Department of Cardiology, Paracelsus Medical University of Salzburg, Salzburg, Austria; 3 Department of Internal Medicine, Cardiology and Nephrology, University Hospital Wiener Neustadt, Wiener Neustadt, Austria; 4 Medical Faculty, Sigmund Freud University, Vienna, Austria; 5 Department of Medicine, Faculty of Medicine and Dentistry, Danube Private University, Krems, Austria; 6 Division of Cardiology, Department of Internal Medicine II, Medical University of Vienna, Vienna, Austria; 7 Department of Cardiology, Kepler University Hospital Linz, Medical Faculty, Johannes Kepler University Linz, Linz, Austria; 8 Department of Internal Medicine III, Cardiology & Angiology, Medical University of Innsbruck, Innsbruck, Austria; 9 Department of Internal Medicine I With Cardiology and Intensive Care, St. Josef Hospital Braunau, Braunau Am Inn, Austria; 10 Clinical Department of Internal Medicine 3, University Hospital St. Poelten, St. Poelten, Austria; 11 Department of Internal Medicine 1, Clinic Cardinal Schwarzenberg, Schwarzach Im Pongau, Austria; 12 Department of Medicine, St. Johann in Tyrol General Hospital, St. Johann in Tirol, Austria; 13 Division of Cardiology, Department of Internal Medicine, State Hospital Graz II, Graz, Austria; 14 Department of Internal Medicine 2, Hospital of the Order of St. Elizabeth, Linz, Austria; 15 Department of Internal Medicine 2 (Cardiology, Intensive Care Medicine), Klinikum Wels-Grieskirchen, Wels, Austria; 16 Division of Cardiology, Department of Internal Medicine V, Favoriten Clinic, Vienna, Austria; 17 3, Medical Department for Cardiology and Intensive Care Medicine, Klinik Ottakring, Vienna, Austria; 18 Ludwig Boltzmann Cluster for Cardiovascular Research, Vienna, Austria; 19 Institute of Vascular Medicine and Cardiac Electrophysiology, Karl Landsteiner Society, 3100 St. Pölten, Austria; 20 Core Facility Clinical Trials Unit, Medical University of Graz, Graz, Austria

**Keywords:** Hypertrophic cardiomyopathy, Registry, Methods, Design, Age, Sex

## Abstract

The diagnosis and management of hypertrophic cardiomyopathy (HCM) is increasingly complex, and systematic evidence remains sparse. The Austrian HCM registry aims to provide representative real-world data addressing gaps in evidence. Here, we report design, methods and results after one year enrolment. The Austrian HCM Registry is an ongoing prospective, multicenter registry including University and community hospitals across Austria. Patients with written informed consent undergo a structured assessment of symptoms, past medical history, family history and the presence of HCM-specific red flags. Clinical data derived from electrocardiogram, echocardiography, laboratory analysis, and genetic testing are collected and entered into a web-based, responsive electronic case report form. Bilateral data transfer agreements ensure legal protection of each site. Between March 2024 and May 2025, 7 University centers and 7 community hospitals from 6 out of 9 Austrian federal states were initiated. Overall, recruitment rate was 2.5 patients per site and month, with University centers and community hospitals contributing 92% and 8%. Among 303 enrolled participants, median age was 60 (25th, 75th percentile; 50, 68), 44% were women. Echocardiography, cardiac magnetic resonance imaging and genetic testing were available in 99%, 86% and 60%, respectively. In this first year, participating sites of the Austrian HCM Registry achieved a consistent and fast recruitment rate. Data availability is high and cohort characteristics align well with other registries. The Austrian HCM Registry will serve as a robust research platform providing contemporary real-world data on epidemiology, diagnosis and management of HCM in a Central European country. **ClinicalTrials.gov identifier.** NCT06368518.

**Supplementary Information:**

The online version contains supplementary material available at 10.1007/s10741-026-10630-6.

## Introduction

Hypertrophic cardiomyopathy (HCM) has a prevalence of 1:200 to 1:500 in the general population and is considered the most common specific cardiomyopathy [1]. Patients are at an increased risk of left ventricular outflow tract obstruction (LVOTO), atrial fibrillation, heart failure and sudden cardiac death (SCD) [2].

The understanding of HCM aetiology and modifiers of disease severity is developing at fast pace. Although HCM has traditionally been regarded as an inherited cardiomyopathy, only approximately 40% of patients are genotype-positive (gen +), with a pathogenic or likely pathogenic variant in a sarcomeric gene causing the hypertrophic phenotype [3, 4], and a positive family history of HCM is present in less than 50% of index patients. Disease-causing variants in non-sarcomeric genes related with syndromic disorders (HCM phenocopies) such as Fabry disease or mitochondriopathies can be identified in up to 5% of adult patients, and up to 20% if pediatric patients are included [3, 5]. In adults with hypertrophic phenotype, however, up to 70% of HCM patients remain gene-elusive after genetic testing (gen-) with increasing evidence pointing towards crucial roles of polygenic risk and comorbidity-mediated myocardial hypertrophy [6–8]. The genetic background of HCM can differ by geographical region, with regional clustering of specific variants leading to distinct phenotypic characteristics [9].

HCM management is complex and demanding, as diagnostic and therapeutic options are rapidly evolving while systematic evidence remains limited [3]. Precise diagnostic tools such as advanced echocardiography, cardiac magnetic resonance imaging (CMR), and genetic testing are becoming ubiquitously available. With the advent of sarcomere modulating drugs, and more tailored HCM therapeutics in the pipeline, with emerging heart failure and anti-arrhythmic drugs and devices, complication management offers the potential to control symptoms and improve prognosis [2, 10]. Randomized controlled trials (RCT) are the prerequisite for introduction of these novel therapeutic concepts, but applicability of RCT results in real world settings is limited due to their high patient selectivity and systematic exclusion of high-risk patients [11, 12]. Therefore, patient registries are an invaluable source of real world data to complement findings from RCTs [13].

Austria is a land-locked country in Central Europe with more than 9 million inhabitants. Based on epidemiological estimations at least 40.000 individuals are anticipated to have HCM in Austria. The Austrian population is characterized by a high migration background from neighbouring countries and the Middle East. Since Austria is a high-income country with universal health care, all medical options for HCM diagnosis and management are available throughout Austria to 99% of its inhabitants, including modern drug and device therapies. Country-specific and international HCM registries have been established worldwide, but Austrian or other Central European HCM populations have not been investigated so far [7, 14–19].

The Austrian HCM registry was launched in 2024 as a prospective multicenter registry integrating both University and community-based HCM referral centers throughout Austria. It was designed to deliver country- and region-specific information about epidemiology, diagnosis and management of HCM enrolling patients with both HCM phenotype and/or HCM genotype. Registry methods and aims are harmonized with the 2023 ESC cardiomyopathy guidelines, covering general characteristics but also disease specific red flags and defined gaps in evidence [3]. Here, we present methods and design of the Austrian HCM registry, and participant characteristics after one year enrolment.

## Methods

### General design

The Austrian HCM Registry is a prospective multicenter patient registry enrolling consecutive outpatients with HCM referred to participating centers (Clinicaltrials.gov identifier NCT06368518). It is an extension of the Graz HCM Registry (EC No. 30–286 ex 17/18) and was approved as a multicenter registry by the lead Ethics Committee of the Medical University of Graz in February 2024. Sites were initiated after approval of local ethics committees, and obtaining bilateral data transfer agreements between legal departments of the lead center (Medical University of Graz) and respective sites. Here, the number of coauthorships per publication is contractually defined. Every site is represented in the steering committee serving as the regulatory authority of the registry by the local Principal Investigator or his/her representative with the competence to approve data contribution to specific research projects. Central project management is conducted by an MD and a study coordinator, both with expertise and training in clinical research management (VS, HT). The project management team coordinates project requests, data exports and amendments to Ethical Committees.

Eligibility criteria are based on European Guidelines on the management of cardiomyopathies [3]. Inclusion criteria comprise increased left ventricular wall thickness not solely explained by abnormal loading conditions, or a positive HCM genotype, and willingness to provide written informed consent (IC). As the implementation of inclusion criterion 2b, which allows the inclusion of genotype-positive and phenotype-negative HCM patients, was amended and approved after the run-in period, in this analysis only patients with a hypertrophic phenotype were included. Patients with confirmed cardiac amyloidosis are excluded. As a consequence, the registry includes both patients with sarcomeric hypertrophy and rare HCM phenocopies, such as Danon’s disease, Anderson Fabry disease, mitochondriopathies, syndromic diseases, and others [3]. Detailed eligibility criteria are given in Tables 1 and 2.

**Table 1:** Eligibility criteria of the Austrian Hypertrophic Cardiomyopathy Registry

Inclusion criteria	Inclusion criteria: 1. Patients admitted to HCM outpatient clinics of participating centers of the HCM Registry 2. One of the following criteria: a) hypertrophic phenotype defined as: Interventricular septal thickness ≥ 13 mm and cardiomyopathy-specific red flags* OR Interventricular septal thickness ≥ 15 mm not explainable by loading conditions OR Interventricular septal thickness ≥ 17 mm** b) positive HCM genotype defined as: Class 4 or 5 variant in one of the following genes: Genes: ABCC9, ACTC1, ACTN2, ALPK3, BAG3, CACNA1C, CAV3, COX15, CRYAB, CSRP3, DES, FHL1, FLNC, FHOD3, FXN, GAA, GLA, YPH2, LAMP2, LDB3, MYBPC3, MYH7, MYL2, MYL3, PLN, PRKAG2, PTPN11, RAF1, RIT1, SLC25A4, TNNC1, TNNI3, TNNT2, TPM1, TRIM63, TTR 3. Willingness and ability to provide signed informed consent form prior to participation in any study-related procedures 4. Age ≥ 14 years
Exclusion criteria	1. Confirmed cardiac amyloidosis

Abbreviations: *HCM* hypertrophic cardiomyopathy

* Cardiomyopathy-specific red flags are listed in Table 2

** Based on the study by Sipola et al., patients with refractory hypertension can be included if septal thickness is 17 mm or more [20]

**Table 2:** Parameters suggesting the presence of HCM or HCM phenocopy if septal thickness is 13 or 14 mm complementing inclusion criterion 2a from Table 1; adapted from [3]

Echocardiography	Reduced left ventricular global longitudinal strain
Cardiac magnetic resonance imaging	Patchy mid-wall LGE pattern in areas of hypertrophy or at the anterior and posterior RV insertion points Posterolateral LGE with concentric LVH Low native T1
Family history	Hypertrophic cardiomyopathy or phenocopy in a first-degree relative Sudden cardiac death in a relative

Abbreviations: *LGE* late-gadolinium enhancement; *LVH* left ventricular hypertrophy; *RV* right ventricular

Pseudonymized patient data are entered in an electronic case report form (eCRF) via a web-based, responsive and customizable platform (Phoenix Clinical Trial Management System, Graz, Austria). All clinical procedures both relating to diagnosis and management at enrolment or during follow-up are left to the discretion of the attending physician. Patients who were enrolled during active treatment with mavacamten were included in this analysis, but clinical parameters from the last Mavacamten-naive visit before treatment initiation were used as baseline. For all other patients, sites were advised to enter data derived from the clinical visit closest to written informed consent as the baseline. Recruitment was anticipated at 1 patient per site and month and a total enrolment of 100 patients per year. In this report, we included all patients recruited between the first patient inclusion on 08th March 2024 and the 15th of May 2025. For this report, central verification of eligibility criteria on individual patient level was conducted by two experienced investigators (VS, NV). Clinical follow-up is at the discretion and local standards of the treating sites and physicians, but will in general follow international and national recommendations [3, 21]. A structured assessment of cardiovascular and non-cardiovascular outcomes will be conducted which will cover all-cause and cause-specific mortality, non-fatal cardiovascular events and incident or worsening cardiovascular disease.

### Procedures

**Fig. 1:**
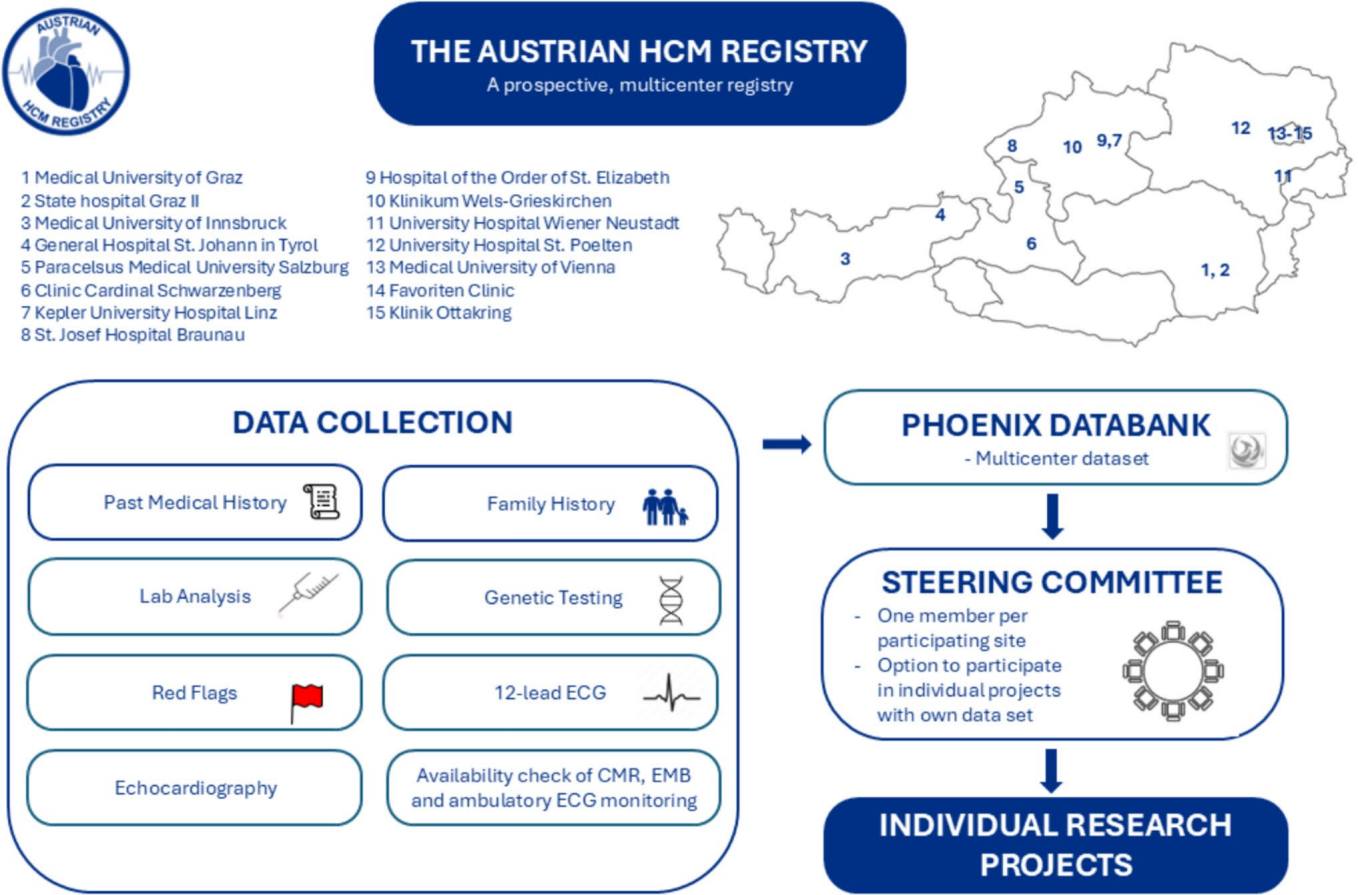
Graphical overview of the design and methods of the Austrian HCM Registry and participating sites

Patients undergo structured examinations including assessment of signs and symptoms of HCM, past medical history including concomitant medication, family history and the presence of HCM-specific red flags as defined in European guidelines (Fig. 1) [3]. Clinical data derived from electrocardiogram, echocardiography, laboratory analysis including N-terminal pro B-type natriuretic peptide (NT-proBNP), and genetic testing are collected focusing on a lean variable dictionary and, in addition, specific hypothesis-driven research parameters. The variable dictionary is provided in Supplementary Table 1.

### Statistical analysis

Continuous variables are given as medians (25th to 75th percentile) or mean ± standard deviation (only in case of normal distribution), categorical variables as absolute frequencies (percentages). To enable assessment of data plausibility and comparisons with other registries, baseline characteristics were stratified according to age categories and sex. Between group comparisons were performed by Fisher’s exact or Chi-square test for categorical variables, and by Mann–Whitney-U or Kruskal–Wallis test for continuous variables, as appropriate. All statistical analyses were performed using SPSS statistical software package (SPSS Inc., Version 30.0, IBM Company, USA). A two-tailed *p*-value < 0.05 was considered statistically significant.

## Results

Between March 2024 and May 2025, all 16 initially contacted centers agreed to participate. In one center, initiation process was halted due to site inactivity, and initiation process of another site was not finalized by May 2025, yielding a total of 14 initiated sites and 1 site with pending initiation by May 2025. The consortium currently is composed of 7 University tertiary care centers (Graz, Innsbruck, Linz, Salzburg, St. Poelten, Vienna, Wiener Neustadt) and 8 community hospitals (Braunau: Graz 2; St. Johann in Tyrol; Linz St. Elisabeth Order; Vienna Favoriten; Vienna Ottakring; Schwarzach; Wels), with 6 out of 9 Austrian federal states being represented (Table 3).

**Table 3:** Participating sites of the Austrian HCM registry

Federal State/Site	Hospital type
Styria	
Medical University of Graz	University tertiary care center
State hospital Graz II	Community hospital
Tyrol	
Medical University of Innsbruck	University tertiary care center
General hospital St. Johann in Tyrol	Community hospital
Salzburg	
Paracelsus Medical University of Salzburg	University tertiary care center
Clinic Cardinal Schwarzenberg	Community hospital
Upper Austria	
Kepler University Hospital Linz	University tertiary care center
St. Josef Hospital Braunau	Community hospital
Hospital of the Order of St. Elizabeth	Community hospital
Klinikum Wels-Grieskirchen	Community hospital
Lower Austria	
University Hospital Wiener Neustadt	University tertiary care center
University Hospital St. Poelten	University tertiary care center
Vienna	
Medical University of Vienna	University tertiary care center
Favoriten Clinic	Community hospital
Klinik Ottakring	Community hospital

**Fig. 2:**
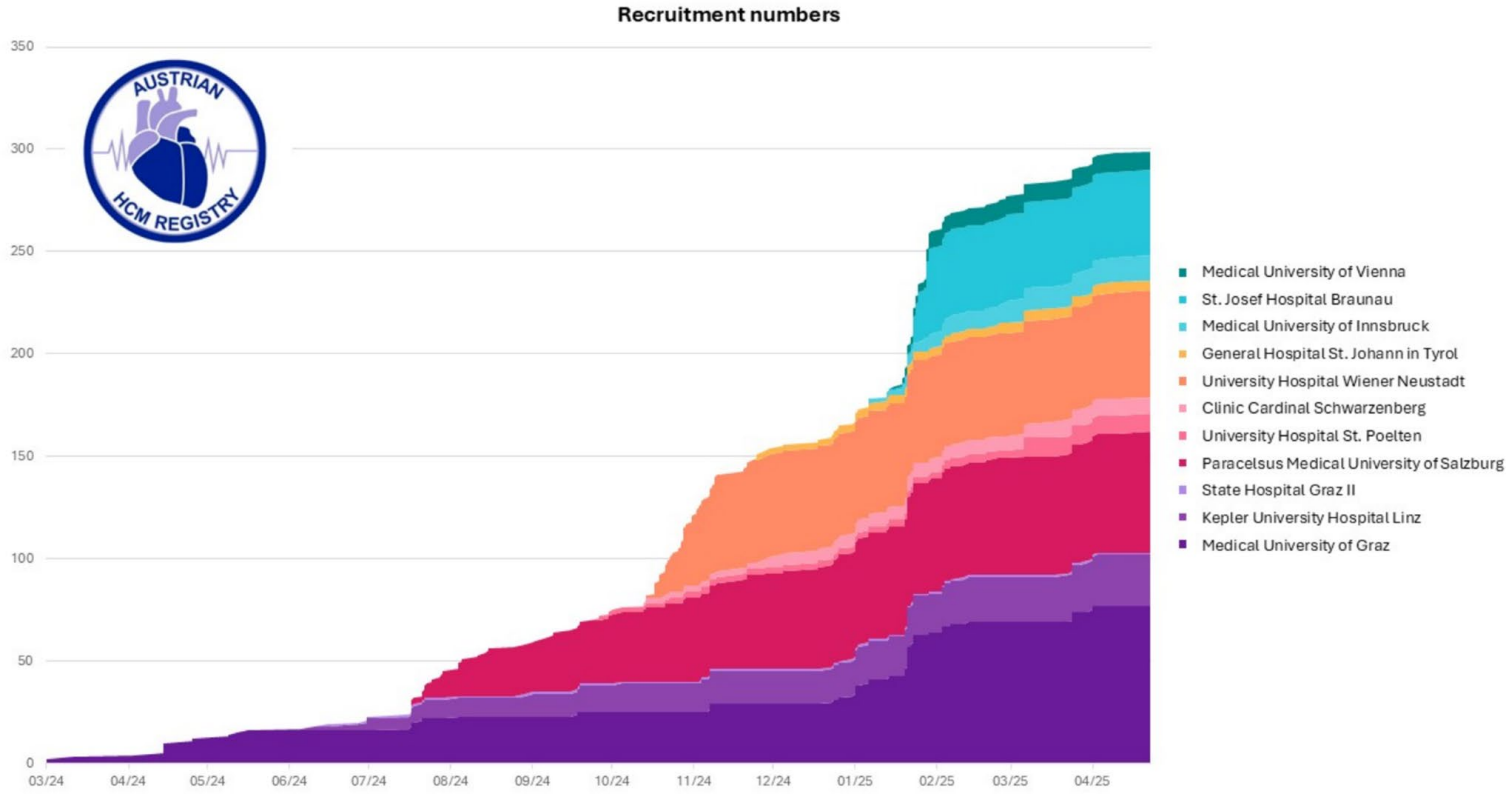
Cumulative recruitment numbers of the Austrian HCM Registry from March 2024 until May 2025 stratified by site

By May 2025, 303 patients with HCM were enrolled (Fig. 2). Inclusions of first patients progressed sequentially. University centers accounted for 92% of total recruitment (280 patients; 4.6 patients per site and month after first patient in), while community hospitals contributed 8% (23 patients; 0.4 patients per center and month after first patient in). Three community hospitals had no patient included. Overall, recruitment rate was 2.5 patients per site and month.

### Performed investigations

The rate of available diagnostic procedure results was overall high (Table 4), with 99% for echocardiography and 86% for CMR at registry enrolment. Red flags were assessed in 99%. Genetic testing was performed in 182 patients (60%) with genetic testing results available in 158 patients (52%). Genotype-positive HCM was detected in 58 patients equivalent to 37% of tested patients and 19% of the total patient sample. Comparing University and community hospitals, conduct of echocardiography (99% vs. 99%, *p* = 0.903) and CMR (86% vs. 85%, *p* = 0.691) did not differ. Investigations to rule out cardiac amyloidosis were less often applied in University hospitals (Tc^99^ bone scintigraphy: 31% vs. 52%, *p* = 0.042; free light chain assessment: 55% vs. 91%, *p* < 0.001). Genetic testing was more often performed in University hospitals (62% vs. 35%, *p* = 0.01).

**Table 4:** Overview of all cardiac investigations performed

Laboratory assessment – n (%)	299 (99%)
ECG – n (%)	299 (99%)
Ambulatory ECG monitoring – n (%)	194 (64%)
Transthoracic echocardiography – n (%)	299 (99%)
CMR – n (%)	260 (86%)
Endomyocardial biopsy – n (%)	10 (3%)
Genetic testing performed* – n (%)	182 (60%)
Red flag assessment – n (%)	299 (99%)

Categorical variables as absolute frequencies (percentages)

Abbreviations: *ECG* electrocardiogram; *CMR* cardiac magnetic resonance tomography

* Genetic testing was performed in 182 patients with test results pending in 24 patients

### Demographics

Mean and median age at investigation were 59 [15] and 61 [52–69] years, respectively, and 133 (44%) were women. Most common symptoms were exertional dyspnoea (NYHA functional class ≥ 2: 67%), palpitations (35%) and angina pectoris (29%), with 21% of participants being asymptomatic. Median MWTH indexed to body surface area (MWTHi) was 11 [9, 12] mm/m^2^, mean LA diameter was 43 [7] mm. Atrial fibrillation was diagnosed in 21% and 9% had an implanted ICD. Median NT-proBNP was 526 pg/ml [232–1300], and LVOT obstruction was prevalent in 60%. The most common comorbidities were arterial hypertension (66%) and hyperlipidaemia (57%). Most commonly prescribed drug classes were beta-blockers (66%) and inhibitors of the renin-angiotensin system (37%). Detailed baseline characteristics are provided in Table 5.

**Table 5:** Baseline characteristics of the overall cohort (*n* = 303)

	Overall (*n* = 303)	n (% miss)
Age at diagnosis – yrs	60 [50, 68]	265 (13%)
Age at investigation – yrs	61 [52, 69]	297 (2%)
Women – n (%)	133 (44)	303 (0%)
Heart rate – bpm	66 [58, 74]	302 (< 1%)
BMI – kg/m^2^	27 [24, 31]	300 (< 1%)
BSA – m^2^	1.93 [1.76, 2.13]	300 (< 1%)
**Ethnicity**		260 (15%)
Caucasian – n (%)	248 (95)	
Others – n (%)	12 (5)	
**Aetiology**		303 (0%)
Genetic testing result available – n (%)	158 (52)	
- Sarcomeric HCM – n (%)	58 (37)	
- Gene-elusive HCM – n (%)	94 (59)	
- Others – n (%)	6 (4)	
HCM without genetic testing/genetic testing result still pending	145 (48)	
**Family history**		
Positive FH for HCM – n (%)	58 (24)	243 (20%)
Positive FH for SCD in a first degree relative – n (%)	17 (7)	248 (19%)
**Comorbidities**		
Arterial hypertension – n (%)	198 (66)	301 (< 1%)
Hyperlipidaemia – n (%)	170 (57)	299 (1%)
Diabetes mellitus – n (%)	57 (19)	300 (< 1%)
Current smoker – n (%)	50 (18)	275 (9%)
Prior smoker – n (%)	61 (22)	275 (9%)
Atrial fibrillation – n (%)	62 (21)	302 (< 1%)
**Previous cardiovascular events**		300 (< 1%)
Stroke – n (%)	19 (6)	
Aborted sudden death – n (%)	6 (2)	
Myocardial infarction – n (%)	19 (6)	
**Procedures prior to enrolment**		303 (0%)
Septal myectomy – n (%)	10 (3)	
Alcohol septal ablation – n (%)	9 (3)	
CIED – n (%)	41 (14)	303 (0%)
Pacemaker – n (%)	15 (5)	
- Conduction System Pacing – n (%)	0 (0)	
- CRT—n (%) *	2 (0.7)	
- Single or dual chamber pacemaker – n (%)	13 (4)	
ICD – n (%)	27 (9)	
- Transvenous ICD – n (%) *	13 (4)	
- Subcutaneous ICD – n (%)	14 (5)	
**Symptoms**		
Asymptomatic – n (%) **	62 (21)	298 (2%)
NYHA Class – n (%)		301 (< 1%)
I	98 (32)	
II	129 (43)	
II-III	41 (13)	
III	32 (11)	
IV	1 (1)	
Angina pectoris – n (%)	87 (29)	301 (< 1%)
Syncope – n (%)	44 (15)	292 (4%)
Palpitations – n (%)	95 (35)	274 (10%)
**Laboratory parameters**		
NT-proBNP – pg/ml	526 [232, 1300]	283 (7%)
Troponin T – pg/ml	16 [10, 25]	244 (19%)
eGFR – ml/min/1,73m^2^	71 [60, 88]	266 (12%)
**Echocardiography**		
LVEF – %	60 [56, 65]	296 (2%)
LAVI – ml/m^2^	40 [32, 51]	188 (40%)
LA diameter—mm	42 [38, 48]	239 (21%)
MWTH – mm	20 [18, 23]	293 (3%)
MWTH indexed to BSA – mm/m^2^	11 [9, 12]	293 (3%)
Septal morphology – n (%)		272 (10%)
Sigmoid	103 (38)	
Reverse curve	91 (33)	
Neutral	63 (23)	
Apical	15 (6)	
LVOTO*** – n (%)	136 (60) ****	228 (25%)
Resting LVOTO – n (%)	82 (36)	
Provocative LVOTO – n (%)	49 (21)	
Resting LVOT gradient – mmHg	14 [7, 43]	
Valsalva LVOT gradient – mmHg	40 [9, 91]	
**Medications**		301 (< 1%)
Beta-blockers – n (%)	198 (66)	
Disopyramide – n (%)	0 (0)	
Verapamil/Diltiazem – n (%)	29 (10)	
ACE-inhibitors – n (%)	33 (11)	
Angiotensin II receptor blockers – n (%)	62 (21)	
Sacubitril/valsartan – n (%)	15 (5)	
Mineralocorticoid receptor antagonists – n (%)	49 (16)	
SGLT2-Inhibitors – n (%)	69 (23)	
Loop diuretics – n (%)	33 (11)	
Antiplatelets – n (%)	69 (23)	
Oral anticoagulants – n (%)	61 (20)	

Continuous variables are given as medians (25th to 75th percentile), categorical variables as absolute frequencies (percentages)

Abbreviations: *ACE* angiotensin-converting enzyme; *BMI* body mass index; *bpm* beats per minute; *BSA* body surface area; *CIED* cardiac implantable electronic device; *CRT* cardiac resynchronization device; *eGFR* estimated glomerular filtration rate; *FH* family history; *ICD* implantable cardioverter defibrillator; *LAVI* left atrial volume index; *LVEF* left ventricular ejection fraction; *LVOTO* left ventricular outflow tract obstruction; *MWTH* maximal wall thickness; *nHCM* non obstructive hypertrophic cardiomyopathy; *NT-proBNP* N-terminal pro-B-type natriuretic peptide; *NYHA* New York Heat Association; *oHCM* obstructive hypertrophic cardiomyopathy; *SCD* sudden cardiac death; *SGLT2* sodium glucose co-transporter 2

* One patient had a CRT-D and was classified into two categories (CRT and transvenous ICD).

** Patients without dyspnea, angina pectoris, prior syncopes and palpitations are counted as asymptomatic

*** LVOT gradients ≥ 30 mmHg are counted as LVOTO

**** Differentiation between resting and provocative LVOTO could not be made in five patients with Valsalva gradient ≥ 30 mmHg, where resting LVOT gradient was not available

Age at diagnosis was available in 265 patients (13% missing), with a mean and median of 57 ± 16 and 60 (50—68) years, respectively. The cohort was stratified according to age at diagnosis of < 40 years (*n* = 40, 15.1%), 40–65 years (*n* = 137, 51.7%), and > 65 years (*n* = 88, 33.2%) (Table 6). Across age categories, there was a significant increase in female sex, comorbidities, NYHA functional class, NT-proBNP, Troponin T and MWTHi, and a decrease in rates of positive genotype, previous SCD, and implanted ICD. Patients < 40 years were characterized by a high proportion of positive genotype (65%), implanted ICD (23%), and men (75%). Patients aged > 65 years were characterized by high rates of comorbidities (arterial hypertension 86%, hyperlipidaemia 64%).

**Table 6:** Baseline characteristics stratified by age at diagnosis (*n* = 265)

	Age < 40 years (*n* = 40, 15%)	Age 40–65 years (*n* = 137, 52%)	Age > 65 years (*n* = 88, 33%)	*P*-value
Women – n (%)	10 (25)	52 (38)	55 (63)	** < 0.001**
BMI – kg/m^2^	26 [23, 32]	28 [25, 31]	25 [23, 30]	**0.005**
BSA – m^2^	1.95 [1.79, 2.22]	1.99 [1.84, 2.15]	1.79 [1.66, 1.95]	** < 0.001**
**Aetiology**				
Genetic testing result available – n (%)	24 (60)	71 (52)	41 (47)	0.163
- Sarcomeric HCM – n (%)	15 (38)	21 (15)	11 (13)	**0.003**
- Gene-elusive HCM – n (%)	8 (20)	46 (34)	30 (34)	0.183
- Others – n (%)	1 (3)	4 (3)	0 (0)	0.201
HCM without genetic testing/genetic testing result still pending	16 (40)	66 (48)	47 (53)	0.163
**Comorbiditie**s				
Arterial hypertension – n (%)	12 (31)	90 (66)	76 (86)	** < 0.001**
Hyperlipidaemia – n (%)	8 (21)	91 (67)	56 (64)	** < 0.001**
Diabetes mellitus – n (%)	1 (3)	30 (22)	23 (26)	**0.007**
Current smoker – n (%)	5 (14)	30 (24)	9 (11)	0.310
Prior smoker – n (%)	7 (19)	28 (22)	19 (23)	0.641
Atrial fibrillation – n (%)	3 (8)	28 (21)	22 (25)	**0.033**
**Previous cardiovascular events**				
Stroke – n (%)	1 (3)	8 (6)	8 (9)	0.325
Aborted sudden death – n (%)	2 (5)	3 (2)	0 (0)	**0.048**
Myocardial infarction – n (%)	0 (0)	8 (6)	9 (10)	0.224
**Procedures prior to enrolment**				
Septal myectomy – n (%)	3 (8)	7 (5)	0 (0)	**0.021**
Alcohol septal ablation – n (%)	1 (3)	4 (3)	3 (3)	0.769
ICD – n (%)	9 (23)	9 (7)	3 (3)	** < 0.001**
**Symptoms**				
Asymptomatic – n (%) *	8 (21)	28 (21)	16 (18)	0.656
NYHA Class – n (%)				** < 0.001**
I	20 (50)	44 (32)	20 (23)	
II	17 (43)	59 (43)	40 (46)	
II-III	1 (3)	22 (16)	11 (13)	
III	2 (5)	11 (8)	16 (18)	
IV	0 (0)	0 (0)	1 (1)	
Angina pectoris – n (%)	8 (20)	47 (35)	22 (25)	0.811
Syncope – n (%)	9 (23)	18 (13)	12 (14)	0.339
Palpitations – n (%)	10 (25)	45 (33)	31 (36)	0.212
**Laboratory parameters**				
NT-proBNP – pg/ml	355 [118, 979]	410 [190, 1052]	724 [334, 1877]	**0.001**
Troponin T – pg/ml	13 [8, 20]	15 [10, 25]	18 [14, 30]	**0.007**
eGFR – ml/min/1,73m^2^	90 [81, 101]	71 [60, 88]	63 [51, 73]	** < 0.001**
**Echocardiography**				
LVEF – %	59 [55, 64]	61 [56, 66]	60 [56, 65]	0.154
LAVI – ml/m^2^	32 [27, 42]	39 [31, 57]	41 [34, 48]	0.124
MWTH – mm	20 [17, 23]	20 [18, 23]	20 [18, 22]	0.753
MWTH indexed to BSA – mm/m^2^	10 [8, 12]	10 [9, 12]	11 [10, 12]	**0.027**
Septal morphology – n (%)				
Sigmoid	9 (23)	52 (38)	34 (39)	0.162
Reverse curve	14 (36)	37 (27)	24 (27)	0.418
Neutral	11 (28)	25 (18)	20 (23)	0.748
Apical	3 (8)	7 (5)	3 (3)	0.304
LVOTO** – n (%)	11 (50)	61 (60)	45 (63)	0.332
Resting LVOTO – n (%)	9 (29)	34 (29)	27 (33)	0.569
Provocative LVOTO – n (%)	2 (9)	25 (25)	15 (22)	0.472

Continuous variables are given as medians (25th to 75th percentile), categorical variables as absolute frequencies (percentages)

Abbreviations: *BMI* body mass index; *BSA* body surface area; *eGFR* estimated glomerular filtration rate; *ICD* implantable cardioverter defibrillator; *LAVI* left atrial volume index; *LVEF* left ventricular ejection fraction; *LVOTO* left ventricular outflow tract obstruction; *MWTH* maximal wall thickness; *NT-proBNP* N-terminal pro-B-type natriuretic peptide; *NYHA* New York Heat Association; *SCD* sudden cardiac death

* Patients without dyspnea, angina pectoris, prior syncopes and palpitations are counted as asymptomatic

** LVOT gradients ≥ 30 mmHg are counted as LVOTO

*** Differentiation between resting and provocative LVOTO could not be made in five patients with Valsalva gradient ≥ 30 mmHg, where resting LVOT gradient was not available

Sex was reported in all patients, with 44% being women and 56% men. Women were significantly older (both age at diagnosis and at investigation), and had a lower BMI. The proportion of positive genotype was higher in women, as were indices of disease severity such as NYHA functional class, NT-proBNP levels, MWTHi, and the proportion of LVOTO (Table 7).

**Table 7:** Baseline characteristics stratified by sex

	Female (*n* = 133)	Male (*n* = 170)	*P*-value
Age at diagnosis – yrs	64 [54, 73]	56 [47, 63]	** < 0.001**
Age at investigation – yrs	66 [58, 73]	57 [49, 65]	** < 0.001**
BMI – kg/m^2^	26 [23, 31]	28 [25, 31]	**0.002**
BSA – m^2^	1.76 [1.64, 1.90]	2.08 [1.92, 2.19]	** < 0.001**
**Aetiology**			
Genetic testing result available – n (%)	67 (50)	91 (54)	0.586
- Sarcomeric HCM – n (%)	32 (24)	26 (15)	0.054
- Gene-elusive HCM – n (%)	31 (23)	63 (37)	**0.010**
- Others – n (%)	4 (3)	2 (1)	0.256
HCM without genetic testing/genetic testing result still pending	66 (50)	79 (47)	0.586
**Comorbidities**			
Arterial hypertension – n (%)	94 (71)	104 (62)	0.111
Hyperlipidaemia – n (%)	85 (64)	85 (52)	**0.028**
Diabetes mellitus – n (%)	27 (21)	30 (18)	0.569
Current smoker – n (%)	27 (23)	23 (15)	0.091
Prior smoker – n (%)	16 (13)	45 (29)	**0.002**
Atrial fibrillation – n (%)	25 (19)	37 (22)	0.508
**Previous cardiovascular events**			
Stroke – n (%)	9 (7)	10 (6)	0.687
Aborted sudden death – n (%)	2 (2)	4 (2)	0.600
Myocardial infarction – n (%)	7 (5)	12 (7)	0.812
**Procedures prior to enrolment**			
Septal myectomy – n (%)	5 (4)	6 (4)	0.915
Alcohol septal ablation – n (%)	7 (5)	2 (1)	**0.038**
ICD – n (%)	10 (8)	17 (10)	0.452
**Symptoms**			
Asymptomatic – n (%) *	17 (13)	45 (27)	**0.003**
NYHA Class – n (%)			** < 0.001**
I	24 (18)	74 (44)	
II	68 (51)	61 (36)	
II-III	21 (16)	20 (12)	
III	18 (14)	14 (8)	
IV	1 (1)	0 (0)	
Angina pectoris – n (%)	39 (29)	48 (28)	0.115
Syncope – n (%)	18 (14)	26 (16)	0.864
Palpitations – n (%)	46 (35)	49 (29)	0.250
**Laboratory parameters**			
NT-proBNP – pg/ml	818 [356, 1857]	336 [148, 939]	** < 0.001**
Troponin T – pg/ml	15 [9, 27]	17 [10, 25]	0.737
eGFR – ml/min/1,73m^2^	64 [55, 81]	81 [61, 90]	** < 0.001**
**Echocardiography**			
LVEF – %	61 [59, 67]	60 [55, 65]	**0.001**
LAVI – ml/m^2^	39 [32, 48]	41 [31, 54]	0.590
MWTH – mm	19 [18, 23]	20 [18, 23]	0084
MWTH indexed to BSA – mm/m^2^	11 [10, 13]	10 [9, 12]	** < 0.001**
Septal morphology – n (%)			0.257
Sigmoid	52 (44)	51 (33)	
Reverse curve	33 (28)	58 (38)	
Neutral	27 (22)	36 (24)	
Apical	7 (6)	8 (5)	
LVOTO** – n (%)	70 (67) ***	66 (53) ***	**0.031**
Resting LVOTO – n (%)	45 (38)	37 (26)	**0.043**
Provocative LVOTO – n (%)	24 (23)	25 (21)	0.681

Continuous variables are given as medians (25th to 75th percentile), categorical variables as absolute frequencies (percentages)

Abbreviations: *BMI* body mass index; *BSA* body surface area; *eGFR* estimated glomerular filtration rate; *ICD* implantable cardioverter defibrillator; *LAVI* left atrial volume index; *LVEF* left ventricular ejection fraction; *LVOTO* left ventricular outflow tract obstruction; *MWTH* maximal wall thickness; *NT-proBNP* N-terminal pro-B-type natriuretic peptide; *NYHA* New York Heat Association; *SCD* sudden cardiac death

* Patients without dyspnea, angina pectoris, prior syncopes and palpitations are counted as asymptomatic

** LVOT gradients ≥ 30 mmHg are counted as LVOTO

*** Differentiation between resting and provocative LVOTO could not be made in five patients with Valsalva gradient ≥ 30 mmHg, where resting LVOT gradient was not available

## Discussion

This is the first report on design, methods and results of the run-in period of the ongoing prospective multicenter Austrian HCM Registry. After approximately one year of prospective patient enrollment, recruitment rate was overall faster than anticipated and availability of conducted procedures was high indicating a high site motivation and efficient registry performance. Moreover, the high availability of diagnostic procedures proves a high quality of patient care at participating centers.

The better than anticipated pace of recruitment and data quality indicates overall high site motivation. Certain measures were implemented in advance to ensure transparent regulation of the scientific use of data and the conduct of registry-based research projects. A per-patient fee is contractually defined and is regularly reimbursed by the Registry lead site upon invoicing. Data transfer agreements were bilaterally negotiated between the legal department of the Medical University Graz and legal departments of respective sites. They specify that every site is represented on the Registry Steering Committee, retains the right to use its own data for scientific purposes, and can also use the registry platform solely for local site-specific research purposes. The contribution of site-specific data to a multicenter project therefore requires prior approval from the respective site/Registry Steering Committee. The numbers of coauthors per publication have been defined and depends on the number of contributed patients to a specific project by the respective site. Central management of registry-based projects (i.e. ethical approval, data export and provision project-specific lead) is coordinated by a central project management team enabling fast and efficient initiation of registry-based projects. This legal framework of the Austrian HCM Registry ensures legal protection and scientific merit for all partners and is likely a key factor contributing to the high recruitment rate and data quality. Yet, University centers contributed the vast majority (> 90%) of enrolled patients which is a commonly observed pattern in national registries [18, 19]. While this can be explained by relatively late initiation of some of these sites during the reported run-in period, this suggests that engagement of Community centers deserves particular motivation strategies. The relatively low recruitment of community hospitals is explainable by a comparably lower number of eligible patients in these non-referral centers but also to infrastructural drawbacks such as lack of dedicated study personnel and other personnel resources for academic efforts. In order to maximize recruitment rates of community hospitals we already implemented site motivation measures such as a regularly newsletter, an annual investigator meeting and a recruitment fee per enrolled patient, but will also stimulate initiation of research projects by community hospitals in the future.

The baseline characteristics, in context with other European registries, provide insights into contemporary demographic trends of HCM in Western countries. Several HCM registry cohorts from Western European countries with similar design have been published during the last 20 years, from Italy (enrolment period 2000 to 2002), Portugal (2013 to 2015) and France (2010 to 2016) [7, 18, 19]. The German TORCH registry is still recruiting, and HCM specific baseline characteristics have not yet been published [22]. Inclusion criteria differed slightly between cohorts (eg. Italy: inclusion of pediatric patients; France: inclusion of amyloidosis patients). The proportion of patients aged > 65 years at diagnosis increased over time, and also the proportion of women tended to increase. Since the Austrian cohort was initiated after approval of the first myosin inhibitor mavacamten, this high proportion of patients diagnosed at advanced age is mostly likely explainable by an increased rate of incidental diagnosis of HCM, in the context of rising awareness of HCM and LVOTO. This notion is supported by a 24% prevalence of provocable LVOTO in the Austrian registry compared to only 4% reported in the Portuguese registry. Interestingly, however, most indices of disease severity have been largely consistent in these registries across the last two decades, including MWTH (all cohorts 20–21 mm) and left atrial end-systolic diameter (all cohorts 41–45 mm). NT-proBNP levels were not reported in any previous national registry cohort so that a more granular comparison of heart failure severity remains elusive. Some variables such as left atrial volume index showed a considerable rate of data missingness with most likely reasons being incomplete source data acquisition during clinical routine and limited resources for data cleaning and data entry. Yet, data completeness und procedure availability are good compared to previous registries: the proportions of conducted echocardiography (99%), CMR (86%), and genetic testing (60%) were all highest in the Austrian registry compared to other European registries, as was the confirmation of a positive genotype related to the overall cohort (23%). In context with rapidly evolving diagnostic and treatment options for HCM, these trends likely reflect improving clinical care for cardiomyopathies in high-income countries with universal health care [23]. National registries differ from large international multicenter registries, where cohorts are composed of large referral center patient populations prone to referral bias. In line with this, HCM patients enrolled in the American-European NHLBI registry, when compared to the Austrian Registry, were meaningfully younger at diagnosis (mean age 49 years compared to 57 years), and patients aged > 65 years had substantially lower median NT-proBNP levels (320 compared to 797 pg/ml) [17]. Similar discrepancies are apparent with regard to the European Cardiomyopathy Pilot Registry and the SHaRe Registry [24, 25]. A systematic comparison of features between the Austrian HCM Registry and other National and referral center registries is shown in Table 8. There was a high proportion of patients aged > 65 years, which matches well with data from the Tufts Medical Center (United States) reporting an increase of elderly patients within their HCM collective over the last decades [23]. In the Austrian HCM Registry, elderly patients were characterized by high rates of comorbidities supporting the concept that non-genetic drivers of hypertrophy are common in HCM and may contribute to disease severity with advancing age [3]. Interestingly, community hospitals more often performed procedures to rule out cardiac amyloidosis, while genetic testing was less often performed. This is in contrast to the French HCM registry, where diagnostic procedures were overall less often applied in so-called non-expert centers [7]. This may point towards a more comprehensive differential diagnostic work-up beyond the use of CMR, and a rather selective use of genetic testing in non-University settings. It may be speculated that University centers place strong confidence in their local CMR expertise to rule out cardiac amyloidosis, a practice which is also supported by Austrian consensus statements on cardiac amyloidosis and HCM [21, 26]. The Austrian HCM Registry extends earlier national registries by providing representative and contemporary real-world data from a Central European country with broad access to all options of modern HCM management, and also reveals practice patterns specific to Austria.

**Table 8:** Comparison of the Austrian HCM Registry with other National and referral center registries

	National registries			Referral center registries	
	Austrian HCM Registry	French Registry “REMY” [7]	Portuguese Registry “Pro-HCM” [18]	SHaRe Registry [27]	NHLBI Registry [17]
Non-sarcomeric HCM included	Yes	Yes	Yes	No	No
Recruitment period	03/2024 – ongoing	01/2010–01/2016	04/2013–04/2015	1960—ongoing	04/2014–04/2017
Sample size, n	303 (05/2025, ongoing)	1431	1042	7268 (2020, ongoing)	2755
Age at diagnosis, years	57 (16)	Na	53 (16)	48 (16)	Na
Age at investigation, years	59 (15)	55 (17)	Na	Na	49 (11)
Women, %	44	31	41	41	29
MWTH (echocardiography), mm	21 (5)	20 (5)	Na	18 (5)	19 (5)
LA diameter, mm	43 (7)	41–45	Na	43 (10)	42 (1)
Gen + sarcomeric HCM, %	18	20	20	28	30
Arterial hypertension, %	66	Na	Na	18	37
Atrial fibrillation, %	21	Na	11	Na	12
LVOT obstruction, %	60	7–10	35	38	18
ICD implantation, %	9	Na	13	Na	Na
NT-proBNP, pg/ml	526 [232, 1300]	Na	Na	Na	Na

Continuous variables are given as means (standard deviation), categorical variables as percentages

Abbreviations: *HCM* hypertrophic cardiomyopathy; *ICD* implantable cardioverter defibrillator; *LA* left atrial; *LVOT* left ventricular outflow tract; *MWTH* maximal wall thickness; *NT-proBNP* N-terminal pro-B-type natriuretic peptide

In the Austrian HCM Registry, significant sex differences were observed. Although a balanced sex distribution should be expected, women accounted only for 44% of the total sample. Interestingly, they had higher age at diagnosis and were more often genotype-positive. Overall, the clinical picture resembled more a HFpEF phenotype compared to men, since women had smaller LV cavities, higher LVEF and increased markers of heart failure severity including echocardiographic indices of filling pressures, NT-proBNP, and NYHA class. LVOT obstruction was also more common in women which is likely explainable by their higher LVEF and smaller LV cavities observed [28]. These results are in line with previous studies indicating that women with HCM compared to men receive their diagnosis later and at a more advanced disease stage translating to a higher risk of HCM-related death [29, 30]. In congruence with earlier studies, women had lower absolute maximal wall thickness, but higher BSA-indexed MWTH compared to men [31–33]. These results add further support to implementing a BSA-indexed cut-off of MWTH for the diagnosis of HCM to facilitate earlier diagnosis of HCM in women and improve their care [32, 33]. The low proportion of women in patients < 40 years may point towards a structural underdiagnosis in young women in Austria and deserves further investigation.

### Limitations and strengths

There are certain limitations inherent to a registry study design. Referral and selection biases cannot be excluded which may influence generalizability. However, 50% of enrolling sites were community-based hospitals, and recruitment was evenly distributed between several University hospitals which may minimize the risk of referral bias. Moreover, the prospective design narrows the risk of selection bias which is often due to retrospective data acquisition. Incompleteness of data is another drawback, although the lean variable dictionary and the web-responsive eCRF were specifically developed to simplify data acquisition and entry to avoid missing data. Since arterial hypertension may in rare cases lead to severe LV hypertrophy, inclusion of very few patients with LV hypertrophy solely due to hypertensive heart disease cannot be ruled out completely. The family relationship between patients was not captured, but this will be amended. The ready availability of NT-proBNP and the high percentages of conducted transthoracic echocardiography, CMR, red flag assessment, and genetic testing are particular strengths enhancing the value of the Austrian HCM Registry.

### Future prospects

Further site initiations are planned to cover the three missing Austrian federal states Burgenland, Carinthia and Vorarlberg. A structured assessment of cardiovascular and non-cardiovascular outcomes will be conducted which will cover all-cause and cause-specific mortality, non-fatal cardiovascular events and incident or worsening cardiovascular disease. Future analyses of the Austrian HCM Registry will investigate genotype–phenotype associations, supportive heart failure therapy, disease courses under specific therapies, and determinants of HCM-specific complications.

## Conclusions

The Austrian HCM Registry will complement other country-specific and international registries and RCT-derived collectives by providing generalizable, contemporary real-world data on epidemiology, diagnosis and management of HCM in a Central European country.

## Supplementary Information

Below is the link to the electronic supplementary material.

Supplementary file1 (DOCX 34 KB)

## Data Availability

Original data are available upon reasonable request.
