# Supplementary material for: The prospective Austrian hypertrophic cardiomyopathy registry – design, methods and results of the run-in period
Source: Heart Fail Rev. 2026 Apr 15;31(1):56. doi: 10.1007/s10741-026-10630-6 (PMC13083411; doi:10.1007/s10741-026-10630-6)
Supplement: Supplementary file 1 — Supplementary file1 (DOCX 34 KB) [file 10741_2026_10630_MOESM1_ESM.docx]

**The prospective Austrian Hypertrophic Cardiomyopathy Registry – Design, Methods and Baseline Characteristics**

**Heart Failure Reviews**

Viktoria Santner^1^, Christina Granitz^2^, Martin R. Grübler^3,4,5^, Daniel Dalos^6^, Christian Reiter^7^, Marc-Michael Zaruba^8^, Johann Auer^9^, Deddo Moertl^10^, Anna Rab^11^, Peter P. Rainer^12^, Gert Waltl^13^, Christian Ebner^14^, Thomas Weber^15^, Diana Bonderman^16^, Stephan Dobner^4,17,18^, Hannah Tuppinger^1^, Viktoria Höller^1^, Nora Schwegel^1^, Klemens Ablasser^1^, Andreas Zirlik^1^, Amelie Graf^2^, Sarah Gharibeh^2^, Johannes Lanzerstorfer^19^, Katharina Wörgötter^4^, Christopher Mann^6^, Shehroz Masood^6^, Christy Meledeth^7^, Clemens Steinwender^7^, Andre Logtenberg^8^, Moritz Messner^8^, Carina Primus^9^, Pia Auersperg^10^, Lydia Mackova^11^, Christof Baurecht^12^, Susanne Winkler^13^, Stephanie Schneiderbauer-Porod^14^, Kathrin Danninger^15^, Silvia Charwat-Resl^16^, Paul Harbich^17,18^, Rene Krenn, Nicolas Verheyen

Corresponding author:

Nicolas Verheyen, MD PhD Associate Professor

Department of Cardiology, University Heart Center Graz, Medical University of Graz, Graz, Austria

Auenbruggerplatz 15, 8036 Graz, Austria

Phone: +43 316 385 30173; Mail: [nicolas.verheyen@medunigraz.at](mailto:nicolas.verheyen@medunigraz.at)

| **Supplementary Table 1. Variable dictionary of the Austrian HCM Registry.** | | |
| --- | --- | --- |
| **Variable name** | **Description** | **Value Type** |
| **Demographics** | | |
| Subject Number |  | Numeric |
| Patient Initials |  | Text |
| Informed consent signed | Consent to participate | Yes/No |
| Registry Inclusion Date | Date of registry inclusion | Date (DD/MM/YYYY) |
| First Diagnosis Date | Date of first HCM diagnosis | Date (DD/MM/YYYY) |
| Date of Birth | Date of Birth | Date (DD/MM/YYYY) |
| Height | Height | Numeric (centimeters) |
| Weight | Weight | Numeric (kilograms) |
| Ethnicity | Ethnic background | Caucasian, Turkish, African, Asian, Other |
| Other Ethnicity | If other ethnicity, specify | Text |
| Etiology | Etiology of HCM | Diagnostics not completed, Gene-elusive HCM (Class 1-3), Sarcomeric HCM (Class 4-5), Fabry Disease, Danon Disease, PRKAG2 Cardiomyopathy, Hemochromatosis, Cardiac amyloidosis. |
| Other etiology | If other etiology, specify | Text |
| Type of Cardiac amyloidosis | If cardiac amyloidosis, specify type | AL/wtATTR/vATTR |
| Sarcomeric HCM | Genetic variants linked to sarcomeric HCM | Text (e.g. MYBPC3) |
| Type of variant | Specific variant of gene mutation | Text (e.g. c.927-9G>A) |
| ACMG Classification | Classification of genetic variant based on ACMG criteria and genetic report | Class 1-5 |
| **Performed investigations** | | |
| Laboratory testing | Laboratory testing performed | Yes/No |
| Date of laboratory testing |  | Date (DD/MM/YYYY) |
| Genetic testing | HCM genetic testing performed | Yes/No |
| Date of genetic panel testing |  | Date (DD/MM/YYYY) |
| ECG | ECG performed | Yes/No |
| Date of ECG |  | Date (DD/MM/YYYY) |
| Ambulatory ECG monitoring | Ambulatory ECG performed | Yes/No |
| Date of ambulatory ECG monitoring |  | Date (DD/MM/YYYY) |
| Echocardiography | Echocardiography performed | Yes/No |
| Date of echocardiography |  | Date (DD/MM/YYYY) |
| CMR | Cardiac magnetic resonance imaging performed | Yes/No |
| Date of CMR |  | Date (DD/MM/YYYY) |
| EMB | Endomyocardial biopsy performed | Yes/No |
| Date of EMB |  | Date (DD/MM/YYYY) |
| TC^99^ bone scintigraphy | TC^99^ bone scintigraphy performed | Yes/No |
| Date of TC^99^ bone scintigraphy |  | Date (DD/MM/YYYY) |
| Immunofixation Electrophoresis + Free light chain assay | Immunofixation electrophoresis + Free light chain assay performed | Yes/No |
| Date of Immunofixation Electrophoresis + Free light chain assay |  | Date (DD/MM/YYYY) |
| Non-cardiac biopsy | Non-cardiac biopsy performed | Yes/No |
| Date of non-cardiac biopsy |  |  |
| **Symptoms** | | |
| Dyspnea | Dyspnea according to NYHA classification | NYHA I, II, II-III, III, IV |
| Angina pectoris |  | Typical/Atypical/No angina pectoris |
| Palpitations |  | Yes/No |
| Syncope | Previous Syncope | Yes/No |
| **Cardiovascular risk factors** | | |
| Diabetes mellitus |  | Yes/No |
| Arterial hypertension |  | Yes/No |
| Hyperlipidemia |  | Yes/No |
| Smoking status |  | Yes/No/Former Smoker |
| Atrial fibrillation or flutter |  | Yes/No |
| Type of atrial fibrillation or flutter |  | Paroxysmal, Persistent, Long-standing persistent, Permanent |
| Competitive athlete |  | Yes active/No/Yes prior competitive athlete |
| **Family history** | | |
| Adoption status | Is the patient adopted? | Yes/No |
| Family history of HCM | Family history of HCM | Yes/No |
| Family member with HCM | If family history for HCM positive, specify family member | Mother, Father, Brother, Sister, Son, Daughter, Other |
| Age at HCM Diagnosis (Family Member) | Age of family member at HCM diagnosis | Age |
| Family history of SCD | Family history of sudden cardiac death | Yes/No |
| Family member with SCD | If family history for SCD positive, specify family member | Mother, Father, Brother, Sister, Son, Daughter, Other |
| Age at SCD of family member | Age of family member at SCD | Age |
| **Prior medical history** | | |
| Previous Stroke |  | Yes/No |
| Previous Myocardial Infarction |  | Yes/No |
| Previous Coronary Stent Implantation |  | Yes/No |
| Previous CABG |  | Yes/No |
| Previous Heart Valve Surgery/Procedure |  | Yes/No |
| Previous Pacemaker Implantation |  | Yes/No |
| Type of implanted Pacemaker |  | Pacemaker, transvenous ICD/subcutaneous ICD/CRT-P/CRT-D/CSP/Other |
| Previous SCD |  | Yes/No |
| Previous Septal Reduction Therapy |  | Yes/No |
| Type of SRT |  | Myectomy/Alcohol septal ablation |
| **Concomitant medication** | | |
| Name of drug |  | Text |
| Active substance of drug |  | Text |
| Dosage of drug |  | Numeric (mg) |
| Frequency | Times per day | Number |
| Total daily dosage |  | Numeric (mg) |
| **Electrocardiogram** | | |
| Heart Rate | Heart rate from ECG | Numeric (bpm) |
| Rhythm |  | Sinus rhythm/Atrial fibrillation/Atrial Flutter |
| PM stimulation | Pacemaker stimulation at ECG | Yes/No |
| PM stimulation type | Type of pacemaker stimulation | Ventricular sensing/ventricular pacing/atrial sensing/atrial pacing |
| PVC | Premature ventricular contraction | Yes/No |
| SVES | Supraventricular extrasystole | Yes/no |
| PQ interval |  | Numeric (ms) |
| AV block | Atrioventricular block | No AV block/AV block 1/AV block 2 Wenckebach/AV bock 2 Mobitz/complete AV block |
| QRS Duration |  | Numeric (ms) |
| Sokolow-Lyon-Index |  | Numeric (mV) |
| Bundle branch block | Type of bundle branch block | No BBB/RSB/RSB + LAHB/RSB + LPHB/LSB/LAHB/LPHB/unspecific complete block/ventricular pacing |
| QT Time |  | Numeric (ms) |
| T Wave Inversion |  | Yes/No |
| Leads with T Wave Inversion |  | Text |
| **Echocardiography** | | |
| Image quality |  | Good/Sufficient/Insufficient |
| Heart rate at Echocardiography |  | Numeric (bpm) |
| Rhythm at Echocardiography |  | Sinus rhythm/Atrial fibrillation |
| LA ES | Left atrial end-systolic diameter | Diameter (mm) |
| LVOT Diameter | Left ventricular outflow tract diameter | Diameter (mm) |
| IVS ED | Interventricular septal end-diastolic diameter | Diameter (mm) |
| LVD ED | Left ventricular diameter end-diastolic | Diameter (mm) |
| PW ED | Posterior wall end-diastolic diameter | Diameter (mm) |
| LVEF | Left ventricular ejection fraction | Percentage (%) |
| LV Volume ED | Left ventricular end-diastolic volume | Volume (ml) |
| LV Volume ES | Left ventricular end-systolic volume | Volume (ml) |
| LAVI | Left atrial volume index | Volume index (mL/m²) |
| E wave | Mitral valve inflow E | Velocity (cm/s) |
| A wave | Mitral valve inflow A | Velocity (cm/s) |
| e´ medial |  | Velocity (cm/s) |
| e´ lateral |  | Velocity (cm/s) |
| Septal phenotype |  | Sigmoid/Reverse curve/Apical/Neutral |
| RV Diameter ED | Right ventricular diameter end-diastolic | Diameter (mm) |
| TAPSE | Tricuspid annular plane systolic excursion | Distance (mm) |
| RA Area (4CH) | Right atrial area in apical four chamber view | Area (cm²) |
| RA Area (2CH) | Right atrial area in apical two chamber view | Area (cm²) |
| Aortic regurgitation |  | No AR/trace AR/mild AR/moderate AR/severe AR |
| Aortic stenosis |  | No AS/mild AS/moderate AS/severe AS |
| AV Vmax | Maximal velocity of aortic valve | Velocity (m/s) |
| AV VTI | Aortic valve velocity time integral | cm |
| AV Pmean | Mean gradient of aortic valve | Gradient (mmHg) |
| MWTH | Maximal wall thickness | Diameter (mm) |
| LVOTO | Left ventricular outflow tract obstruction | None/Provocable obstruction/Resting obstruction |
| Pmax LVOT resting | Maximum resting LVOT gradient | Gradient (mmHg) |
| Pmax LVOT Valsalva | Maximum LVOT gradient after Valsalva maneuver | Gradient (mmHg) |
| Pmax LVOT Maximal provoced | Maximum LVOT gradient maximal provoked | Gradient (mmHg) |
| Midventricular obstruction | Midventricular obstruction | None/Provocable obstruction/Resting obstruction |
| Pmax midventricular resting | Maximum resting midventricular gradient | Gradient (mmHg) |
| Pmax midventricular Valsalva | Maximum midventricular gradient after Valsalva maneuver | Gradient (mmHg) |
| Pmax midventricular Maximal provoced | Maximum midventricular gradient maximal provoced | Gradient (mmHg) |
| SAM | Systolic anterior motion | Complete SAM/Incomplete SAM |
| Mitral regurgitation |  | No MR/trace MR/mild MR/moderate MR/severe MR |
| Mitral stenosis |  | No MS/mild MS/moderate MS/severe MS |
| Tricuspid regurgitation |  | No TR/trace TR/mild TR/moderate TR/severe TR |
| TR Vmax | Maximal velocity of tricuspid regurgitation | Velocity (m/s) |
| RV free wall | Right ventricular free wall | Diameter (mm) |
| VCI diameter | Vena cava inferior diameter | Diameter (mm) |
| Pericardial effusion |  | Yes/No |
| **Laboratory parameters** | | |
| NT-proBNP | N-terminal pro-B-type natriuretic peptide | Numeric (pg/ml) |
| Hs-TropT | High sensitive Troponin T | Numeric (pg/ml) |
| Hs-TropI | High sensitive Troponin I | Numeric (pg/ml) |
| Serum Creatinine |  | Numeric (mg/dl) |
| eGFR | Estimated Glomerular Filtration Rate | Numeric (ml/min/1.73 m²) |
| hsCRP | High-sensitive C-reactive protein | Numeric (mg/l) |
| CK | Creatine Kinase | Numeric (U/l) |
| HbA1c | Hemoglobin A1c | Percentage (%) |
| Urine Albumin |  | Numeric (mg/l) |
| Urine Creatinine |  | Numeric (mg/dl) |
| Urine Protein |  | Numeric (mg/l) |
| **Red Flags** | | |
| Hearing Aids |  | Yes/No |
| Cataract |  | Yes/No |
| Gait Disturbance |  | Yes/No |
| Polyneuropathy |  | Yes/No |
| Neuropathic Pain |  | Yes/No |
| Peripheral Muscle Diseases |  | Yes/No |
| Learning difficulties |  | Yes/No |
| Carpal Tunnel Syndrome |  | Yes/No |
| Angiokeratomas |  | Yes/No |
| Proteinuria |  | Yes/No |
| Lentigines/Café au lait |  | Yes/No |
| **Abbreviations:** ACMG=American College of Medical Genetics and Genomics; AL=light chain amyloidosis; AR=aortic regurgitation; AS=aortic stenosis; AV=aortic valve; bpm=beats per minute; CABG=coronary artery bypass graft; CMR=cardiac magnetic resonance imaging; CRT=cardiac resynchronization device; ECG=electrocardiogram; eGFR=estimated glomerular filtration rate; EMB=endomyocardial biopsy; FH=family history; HbA1c=hemoglobin A1c; HCM=hypertrophic cardiomyopathy; ICD=implantable cardioverter defibrillator; IVS ED=interventricular septum end-diastolic diameter; LA ES=left atrial end-systolic diameter; LAVI=left atrial volume index; LVD ED=Left Ventricular Diameter end-diastolic; LVEF=left ventricular ejection fraction; LVOTO=left ventricular outflow tract obstruction; MR=mitral regurgitation; MS=mitral stenosis; MWTH=maximal wall thickness; NT-proBNP=N-terminal pro-B-type natriuretic peptide; NYHA=New York Heat Association; PM=pacemaker; PVC=premature ventricular contraction; PW ED=Posterior Wall end-diastolic diameter; RA=right atrial; RV=right ventricular; SAM=systolic anterior motion; SCD=sudden cardiac death; SRT=septal reduction therapy; TAPSE=Tricuspid Annular Plane Systolic Excursion; TR=tricuspid regurgitation; vATTR=hereditary transthyretin amyloid cardiomyopathy; VCI=vena cava inferior; wtATTR=wildtype transthyretin amyloid cardiomyopathy. | | |
